# A pneumonia outbreak associated with a new coronavirus of probable bat origin

**DOI:** 10.1038/s41586-020-2012-7

**Published:** 2020-02-03

**Authors:** Peng Zhou, Xing-Lou Yang, Xian-Guang Wang, Ben Hu, Lei Zhang, Wei Zhang, Hao-Rui Si, Yan Zhu, Bei Li, Chao-Lin Huang, Hui-Dong Chen, Jing Chen, Yun Luo, Hua Guo, Ren-Di Jiang, Mei-Qin Liu, Ying Chen, Xu-Rui Shen, Xi Wang, Xiao-Shuang Zheng, Kai Zhao, Quan-Jiao Chen, Fei Deng, Lin-Lin Liu, Bing Yan, Fa-Xian Zhan, Yan-Yi Wang, Geng-Fu Xiao, Zheng-Li Shi

**Affiliations:** 1 CAS Key Laboratory of Special Pathogens, Wuhan Institute of Virology, Center for Biosafety Mega-Science, Chinese Academy of Sciences, Wuhan, China; 2 Wuhan Jin Yin-Tan Hospital, Wuhan, China; 3 University of Chinese Academy of Sciences, Beijing, China; 4 Hubei Provincial Center for Disease Control and Prevention, Wuhan, China

**Keywords:** Pathogens, Virology, SARS-CoV-2

## Abstract

Since the outbreak of severe acute respiratory syndrome (SARS) 18 years ago, a large number of SARS-related coronaviruses (SARSr-CoVs) have been discovered in their natural reservoir host, bats^1–4^. Previous studies have shown that some bat SARSr-CoVs have the potential to infect humans^5–7^. Here we report the identification and characterization of a new coronavirus (2019-nCoV), which caused an epidemic of acute respiratory syndrome in humans in Wuhan, China. The epidemic, which started on 12 December 2019, had caused 2,794 laboratory-confirmed infections including 80 deaths by 26 January 2020. Full-length genome sequences were obtained from five patients at an early stage of the outbreak. The sequences are almost identical and share 79.6% sequence identity to SARS-CoV. Furthermore, we show that 2019-nCoV is 96% identical at the whole-genome level to a bat coronavirus. Pairwise protein sequence analysis of seven conserved non-structural proteins domains show that this virus belongs to the species of SARSr-CoV. In addition, 2019-nCoV virus isolated from the bronchoalveolar lavage fluid of a critically ill patient could be neutralized by sera from several patients. Notably, we confirmed that 2019-nCoV uses the same cell entry receptor—angiotensin converting enzyme II (ACE2)—as SARS-CoV.

## Abstract (web summary)

Characterization of full-length genome sequences from patients infected with a new coronavirus (2019-nCoV) shows that the sequences are nearly identical and indicates that the virus is related to a bat coronavirus.

## Main

Coronaviruses have caused two large-scale pandemics in the past two decades, SARS and Middle East respiratory syndrome (MERS)^8,9^. It has generally been thought that SARSr-CoV—which is mainly found in bats—could cause a future disease outbreak^10,11^. Here we report on a series of cases caused by an unidentified pneumonia disease outbreak in Wuhan, Hubei province, central China. This disease outbreak—which started from a local seafood market—has grown substantially to infect 2,761 people in China, is associated with 80 deaths and has led to the infection of 33 people in 10 additional countries as of 26 January 2020^12^. Typical clinical symptoms of these patients are fever, dry cough, breathing difficulties (dyspnoea), headache and pneumonia. Disease onset may result in progressive respiratory failure owing to alveolar damage (as observed by transverse chest computerized-tomography images) and even death. The disease was determined to be caused by virus-induced pneumonia by clinicians according to clinical symptoms and other criteria, including a rise in body temperature, decreases in the number of lymphocytes and white blood cells (although levels of the latter were sometimes normal), new pulmonary infiltrates on chest radiography and no obvious improvement after treatment with antibiotics for three days. It appears that most of the early cases had contact history with the original seafood market; however, the disease has now progressed to be transmitted by human-to-human contact.

**Fig. 1:**
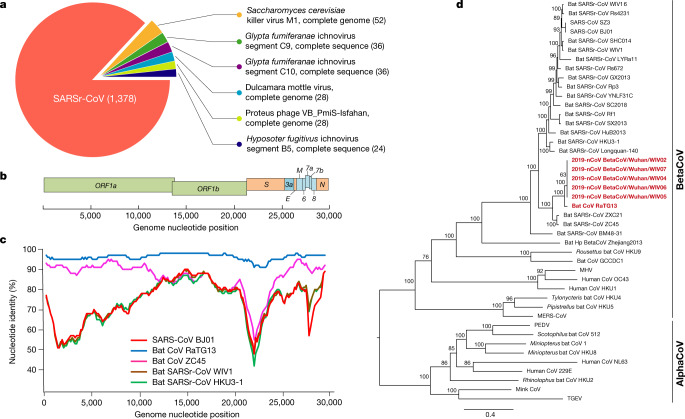
Genome characterization of 2019-nCoV. **a**, Metagenomics analysis of next-generation sequencing of BALF from patient ICU06. **b**, Genomic organization of 2019-nCoV WIV04. M, membrane. **c**, Similarity plot based on the full-length genome sequence of 2019-nCoV WIV04. Full-length genome sequences of SARS-CoV BJ01, bat SARSr-CoV WIV1, bat coronavirus RaTG13 and ZC45 were used as reference sequences. **d**, Phylogenetic tree based on nucleotide sequences of complete genomes of coronaviruses. MHV, murine hepatitis virus; PEDV, porcine epidemic diarrhoea virus; TGEV, porcine transmissible gastroenteritis virus.The scale bars represent 0.1 substitutions per nucleotide position. Descriptions of the settings and software that was used are included in the Methods.

**Extended Data Fig. 1:**
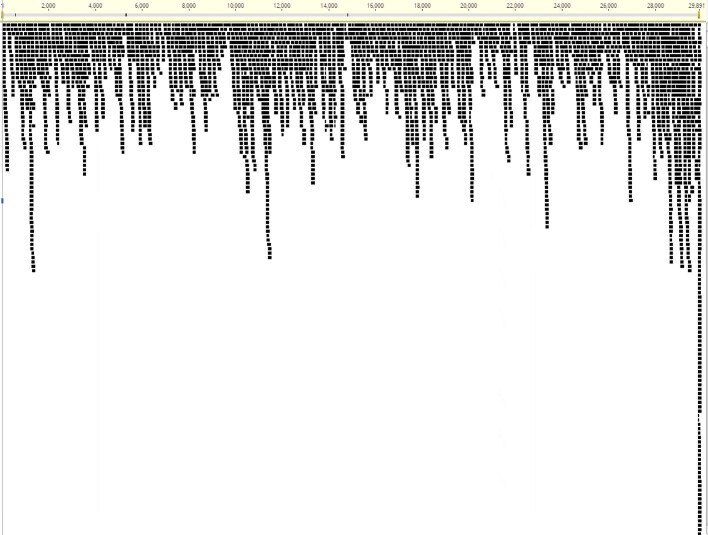
NGS raw reads of sample WIV04 mapping to the 2019-nCoV sequence. The *x* axis indicates the genome nucleotide position and the *y* axis represents the read depth of the mapping.

Samples from seven patients with severe pneumonia (six of whom are sellers or deliverymen from the seafood market), who were admitted to the intensive care unit of Wuhan Jin Yin-Tan Hospital at the beginning of the outbreak, were sent to the laboratory at the Wuhan Institute of Virology (WIV) for the diagnosis of the causative pathogen (Extended Data Table 1). As a laboratory investigating CoV, we first used pan-CoV PCR primers to test these samples^13^, given that the outbreak occurred in winter and in a market—the same environment as SARS infections. We found five samples to be PCR-positive for CoVs. One sample (WIV04), collected from the bronchoalveolar lavage fluid (BALF), was analysed by metagenomics analysis using next-generation sequencing to identify potential aetiological agents. Of the 10,038,758 total reads—of which 1,582 total reads were retained after filtering of reads from the human genome—1,378 (87.1%) sequences matched the sequence of SARSr-CoV (Fig. 1a). By de novo assembly and targeted PCR, we obtained a 29,891-base-pair CoV genome that shared 79.6% sequence identity to SARS-CoV BJ01 (GenBank accession number AY278488.2). High genome coverage was obtained by remapping the total reads to this genome (Extended Data Fig. 1). This sequence has been submitted to GISAID (https://www.gisaid.org/) (accession number EPI_ISL_402124). Following the name given by the World Health Organization (WHO), we tentatively call it novel coronavirus 2019 (2019-nCoV). Four more full-length genome sequences of 2019-nCoV (WIV02, WIV05, WIV06 and WIV07) (GISAID accession numbers EPI_ISL_402127–402130) that were more than 99.9% identical to each other were subsequently obtained from four additional patients using next-generation sequencing and PCR (Extended Data Table 2).

The virus genome consists of six major open-reading frames (ORFs) that are common to coronaviruses and a number of other accessory genes (Fig. 1b). Further analysis indicates that some of the 2019-nCoV genes shared less than 80% nucleotide sequence identity to SARS-CoV. However, the amino acid sequences of the seven conserved replicase domains in ORF1ab that were used for CoV species classification were 94.4% identical between 2019-nCoV and SARS-CoV, suggesting that the two viruses belong to the same species, SARSr-CoV.

**Extended Data Fig. 2:**
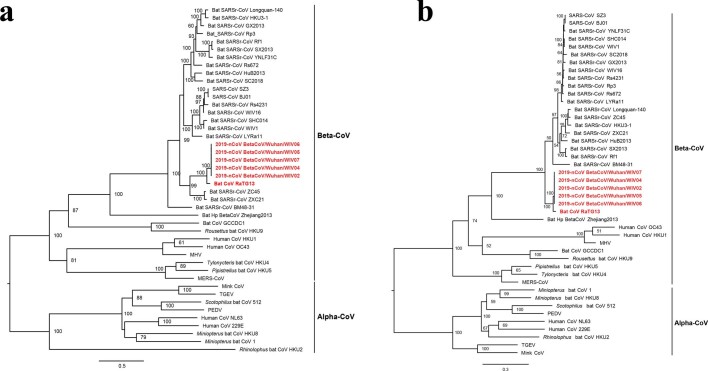
Phylogenetic trees based on the complete S and RdRp gene sequences of coronaviruses. **a**, **b**, Phylogenetic trees on the basis of the gene sequences of *S* (**a**) and *RdRp* (**b**) are shown. 2019-nCoV and bat CoV RaTG13 are shown in bold and in red. The trees were constructed using the maximum likelihood method using the GTR + G substitution model with bootstrap values determined by 1,000 replicates. Bootstraps values of more than 50% are shown.

**Extended Data Fig. 3:**
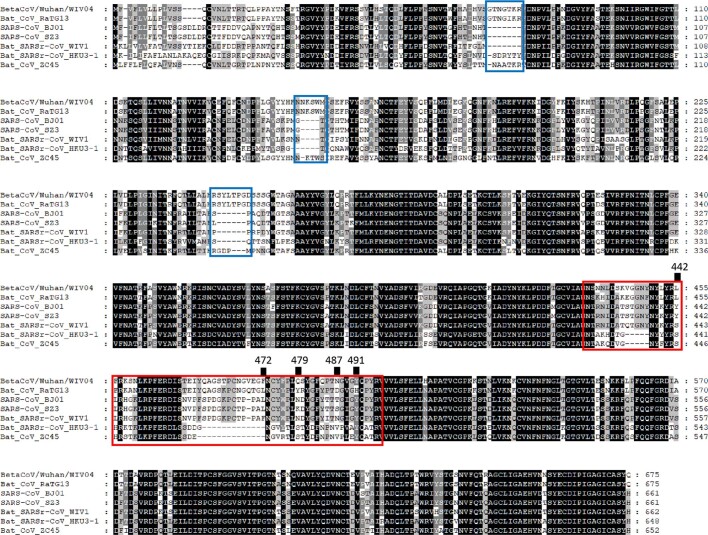
Amino acid sequence alignment of the S1 protein of the 2019-nCoV to SARS-CoV and selected bat SARSr-CoVs. The receptor-binding motif of SARS-CoV and the homologous region of other coronaviruses are indicated by the red box. The key amino acid residues involved in the interaction with human ACE2 are numbered at the top of the aligned sequences. The short insertions in the N-terminal domain of the 2019-nCoV are indicated by the blue boxes. Bat CoV RaTG13 was obtained from *R. affinis*, found in Yunnan province. Bat CoV ZC45 was obtained from *R. sinicus*, found in Zhejiang province.

We then found that a short region of RNA-dependent RNA polymerase (RdRp) from a bat coronavirus (BatCoV RaTG13)—which was previously detected in *Rhinolophus affinis* from Yunnan province—showed high sequence identity to 2019-nCoV. We carried out full-length sequencing on this RNA sample (GISAID accession number EPI_ISL_402131). Simplot analysis showed that 2019-nCoV was highly similar throughout the genome to RaTG13 (Fig. 1c), with an overall genome sequence identity of 96.2%. Using the aligned genome sequences of 2019-nCoV, RaTG13, SARS-CoV and previously reported bat SARSr-CoVs, no evidence for recombination events was detected in the genome of 2019-nCoV. Phylogenetic analysis of the full-length genome and the gene sequences of *RdRp* and *spike* (*S*) showed that—for all sequences—RaTG13 is the closest relative of 2019-nCoV and they form a distinct lineage from other SARSr-CoVs (Fig. 1d and Extended Data Fig. 2). The receptor-binding spike protein encoded by the *S* gene was highly divergent from other CoVs (Extended Data Fig. 2), with less than 75% nucleotide sequence identity to all previously described SARSr-CoVs, except for a 93.1% nucleotide identity to RaTG13 (Extended Data Table 3). The *S* genes of 2019-nCoV and RaTG13 are longer than other SARSr-CoVs. The major differences in the sequence of the *S* gene of 2019-nCoV are the three short insertions in the N-terminal domain as well as changes in four out of five of the key residues in the receptor-binding motif compared with the sequence of SARS-CoV (Extended Data Fig. 3). Whether the insertions in the N-terminal domain of the S protein of 2019-nCoV confer sialic-acid-binding activity as it does in MERS-CoV needs to be further studied. The close phylogenetic relationship to RaTG13 provides evidence that 2019-nCoV may have originated in bats.

**Fig. 2:**
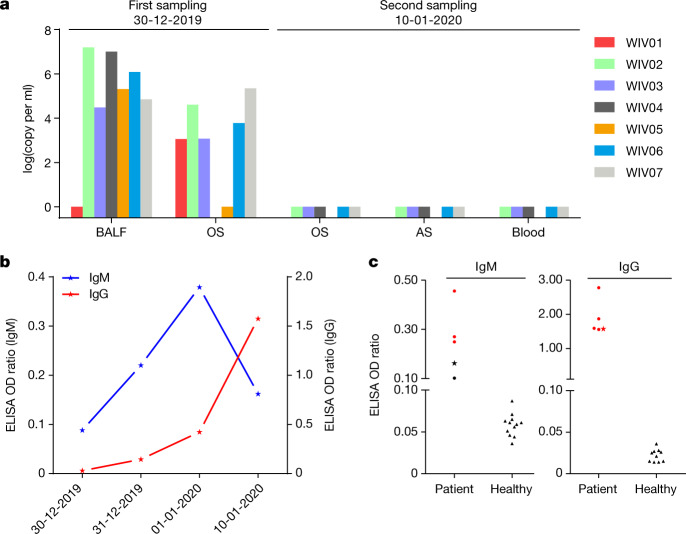
Molecular and serological investigation of patient samples. **a**, Molecular detection of 2019-nCoV in seven patients. Patient information can be found in Extended Data Tables 1, 2. Detection methods are described in the Methods. AS, anal swab; OS, oral swab. **b**, Dynamics of 2019-nCoV antibody levels in one patient who showed signs of disease on 23 December 2019 (ICU-06). OD ratio, optical density at 450–630 nm. The right and left *y* axes indicate ELISA OD ratios for IgM and IgG, respectively. **c**, Serological test of 2019-nCoV antibodies in five patients (Extended Data Table 2). The asterisk indicates data collected from patient ICU-06 on 10 January 2020. **b**, **c**, The cut-off was to 0.2 for the IgM analysis and to 0.3 for the IgG analysis, according to the levels of healthy controls.

**Extended Data Fig. 4:**
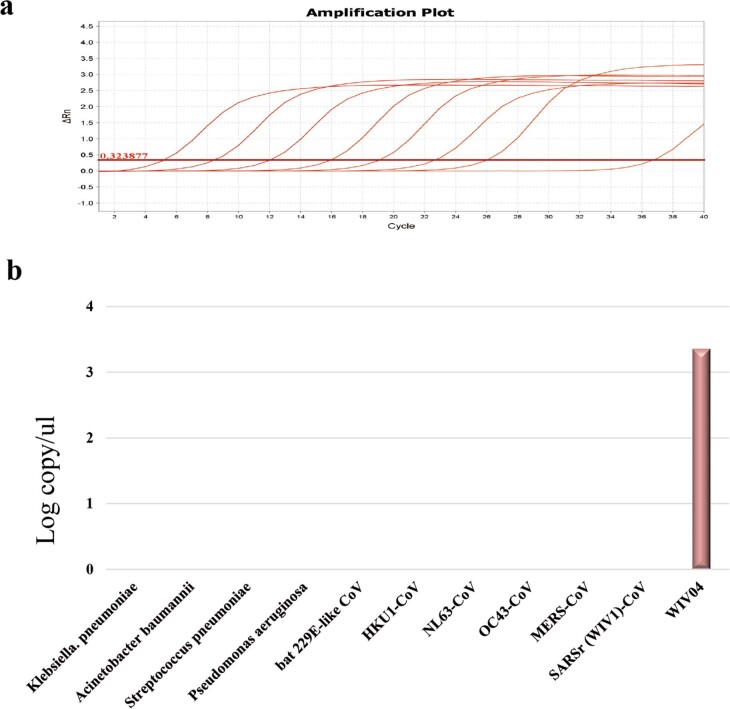
Molecular detection method used to detect 2019-nCoV. **a**, Standard curve for qPCR primers. The PCR product of the *S* gene that was serial diluted in the range of 10^8^ to 10^1^ (lines from left to right) was used as a template. Primer sequences and experimental conditions are described in the Methods. **b**, Specificity of the qPCR primers. Nucleotide samples from the indicated pathogens were used.

We rapidly developed a qPCR-based detection method on the basis of the sequence of the receptor-binding domain of the *S* gene, which was the most variable region of the genome (Fig. 1c). Our data show that the primers could differentiate 2019-nCoV from all other human coronaviruses including bat SARSr-CoV WIV1, which shares 95% identity with SARS-CoV (Extended Data Fig. 4a, b). Of the samples obtained from the seven patients, we found that six BALF and five oral swab samples were positive for 2019-nCoV during the first sampling, as assessed by qPCR and conventional PCR. However, we could no longer detect virus-positive samples in oral swabs, anal swabs and blood samples taken from these patients during the second sampling (Fig. 2a). However, we recommend that other qPCR targets, including the *RdRp* or *envelope* (*E*) genes are used for the routine detection of 2019-nCoV. On the basis of these findings, we propose that the disease could be transmitted by airborne transmission, although we cannot rule out other possible routes of transmission, as further investigation, including more patients, is required.

**Extended Data Fig. 5:**
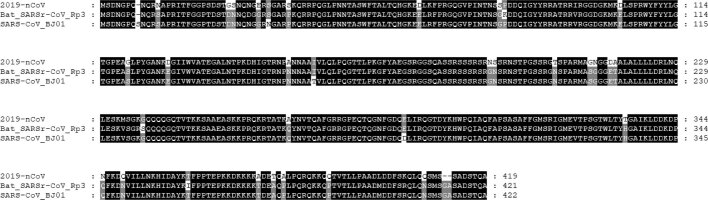
Amino acid sequence alignment of the nucleocapsid protein of 2019-nCoV to bat SARSr-CoV Rp3 and SARS-CoV BJ01. Bat SARSr-CoV Rp3 was obtained from *R. sinicus*, which is found in Guangxi province.

For serological detection of 2019-nCoV, we used a previously developed nucleocapsid (N) protein from bat SARSr-CoV Rp3 as antigen for IgG and IgM enzyme-linked immunosorbent assays (ELISAs), as this protein shared 92% amino acid identity to N protein of 2019-nCoV (Extended Data Fig. 5) and showed no cross-reactivity against other human coronaviruses except SARSr-CoV^7^. We were only able to obtain five serum samples from the seven patients with viral infections. We monitored viral antibody levels in one patient (ICU-06) 7, 8, 9 and 18 days after the onset of disease (Extended Data Table 2). A clear trend was observed in the IgG and IgM titres, which increased over time, except that the IgM titre was decreased in the last sample (Fig. 2b). As a second analysis, we tested samples from 5 of the 7 virus-positive patients around 20 days after disease onset for the presence of viral antibodies (Extended Data Tables 1, 2). All patient samples—but not samples from healthy individuals—were strongly positive for viral IgG (Fig. 2b). There were also three IgM-positive samples, indicating an acute infection.

**Extended Data Fig. 6:**
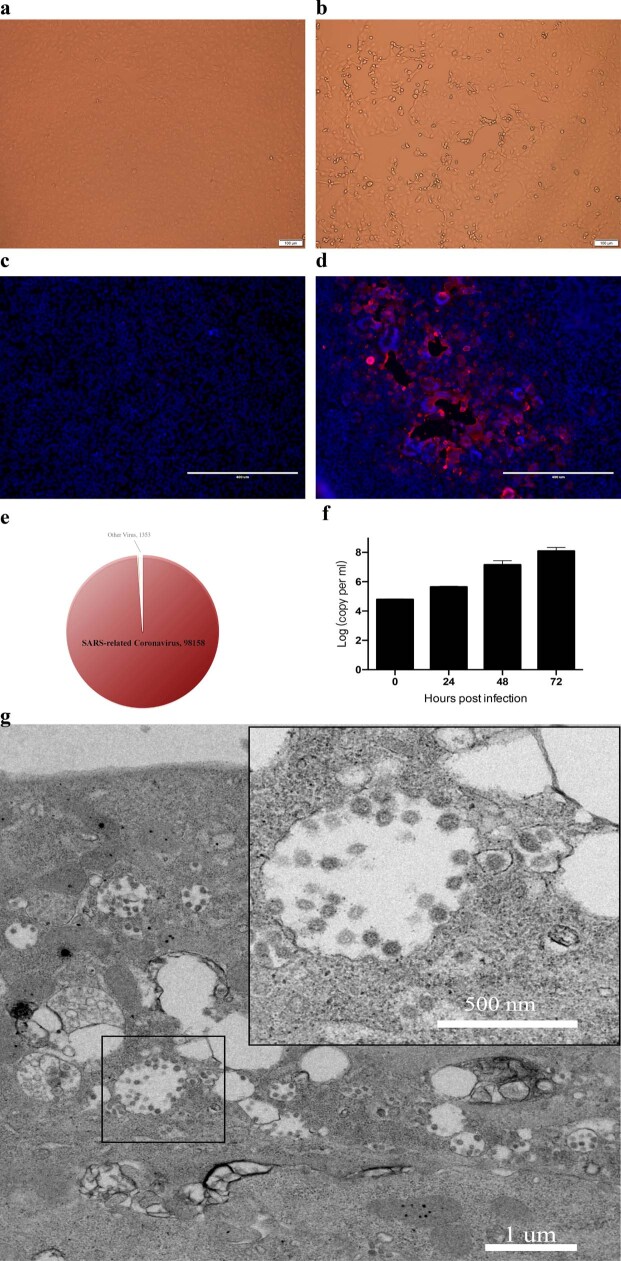
Isolation and antigenic characterization of 2019-nCoV. **a**, **b**, Vero E6 cells are shown at 24 h after infection with mock virus (**a**) or 2019-nCoV (**b**). **c**, **d**, Mock-virus-infected (**c**) or 2019-nCoV-infected (**d**) samples were stained with rabbit serum raised against recombinant SARSr-CoV Rp3 N protein (red) and DAPI (blue). The experiment was conducted twice independently with similar results. **e**, The ratio of the number of reads related to 2019-nCoV among the total number of virus-related reads in metagenomics analysis of supernatants from Vero E6 cell cultures. **f**, Virus growth in Vero E6 cells. **g**, Viral particles in the ultrathin sections were imaged using electron microscopy at 200 kV. The sample was from virus-infected Vero E6 cells. The inset shows the viral particles in an intra-cytosolic vacuole.

We next successfully isolated the virus (called 2019-nCoV BetaCoV/Wuhan/WIV04/2019) from both Vero E6 and Huh7 cells using the BALF sample of patient ICU-06. Clear cytopathogenic effects were observed in cells after incubation for three days (Extended Data Fig. 6a, b). The identity of the strain WIV04 was verified in Vero E6 cells by immunofluorescence microscopy using the cross-reactive viral N antibody (Extended Data Fig. 6c, d) and by metagenomics sequencing, most of the reads of which mapped to 2019-nCoV, and qPCR analysis showed that the viral load increased from day 1 to day 3 (Extended Data Fig. 6e, f). Viral particles in ultrathin sections of infected cells displayed a typical coronavirus morphology, as visualized by electron microscopy (Extended Data Fig. 6g). To further confirm the neutralization activity of the viral IgG-positive samples, we conducted serum-neutralization assays in Vero E6 cells using the five patient sera that were IgG-positive. We demonstrate that all samples were able to neutralize 100 TCID_50_ (50% tissue-culture-infective dose) of 2019-nCoV at a dilution of 1:40–1:80. We also show that this virus could be cross-neutralized by horse anti-SARS-CoV serum (gift from L.-F. Wang) at dilutions of 1:40; however, the potential for cross-reactivity with SARS-CoV antibodies needs to be confirmed with anti-SARS-CoV serum from humans (Extended Data Table 4).

**Fig. 3:**
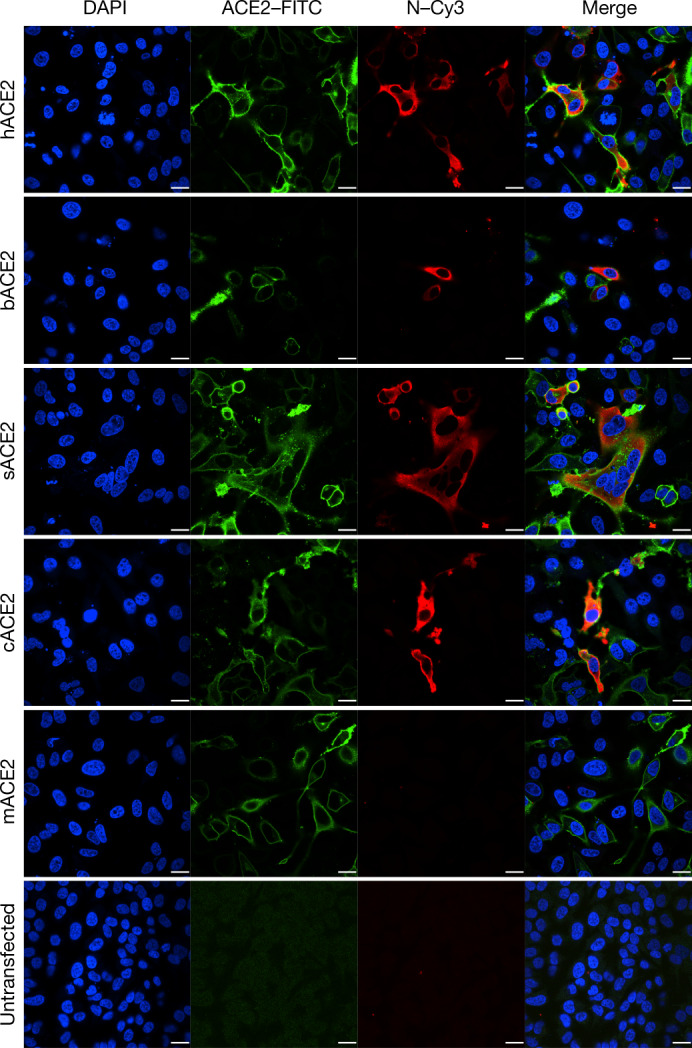
Analysis of the receptor use of 2019-nCoV. Determination of virus infectivity in HeLa cells that expressed or did not express (untransfected) ACE2. The expression of ACE2 plasmid with S tag was detected using mouse anti-S tag monoclonal antibody. hACE2, human ACE2; bACE2, ACE2 of *Rhinolophus sinicus* (bat); cACE2, civet ACE2; sACE2, swine ACE2 (pig); mACE2, mouse ACE2. Green, ACE2; red, viral protein (N); blue, DAPI (nuclei). Scale bars, 10 μm.

**Extended Data Fig. 7:**
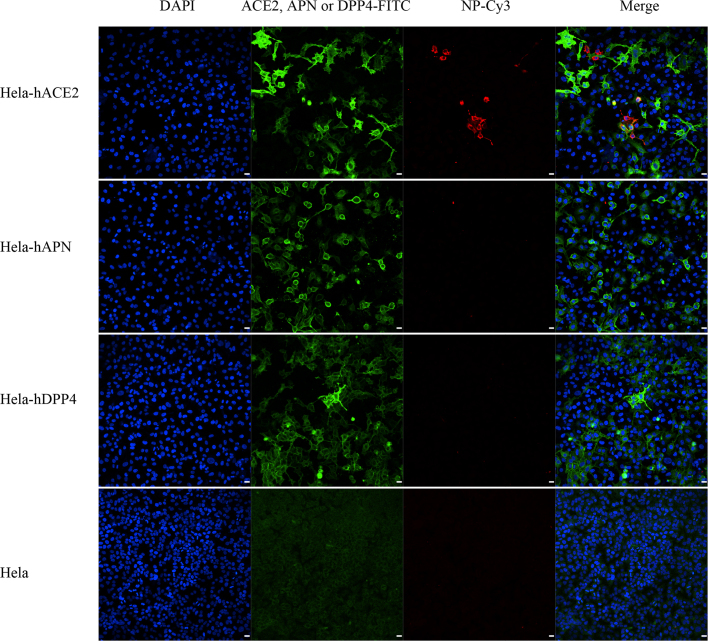
Analysis of 2019-nCoV receptor usage. Determination of virus infectivity in HeLa cells with or without the expression of human APN and DPP4. The expression of ACE2, APN and DPP4 plasmids with S tag were detected using mouse anti-S tag monoclonal antibody. ACE2, APN and DPP4 proteins (green), viral protein (red) and nuclei (blue) are shown. Scale bars, 10 μm.

ACE2 is known to be a cell receptor for SARS-CoV^14^. To determine whether 2019-nCoV also uses ACE2 as a cellular entry receptor, we conducted virus infectivity studies using HeLa cells that expressed or did not express ACE2 proteins from humans, Chinese horseshoe bats, civets, pigs and mice. We show that 2019-nCoV is able to use all ACE2 proteins, except for mouse ACE2, as an entry receptor to enter ACE2-expressing cells, but not cells that did not express ACE2, indicating that ACE2 is probably the cell receptor through which 2019-nCoV enters cells (Fig. 3). We also show that 2019-nCoV does not use other coronavirus receptors, such as aminopeptidase N (APN) and dipeptidyl peptidase 4 (DPP4) (Extended Data Fig. 7).

The study provides a detailed report on 2019-nCoV, the likely aetiological agent responsible for the ongoing epidemic of acute respiratory syndrome in China and other countries. Virus-specific nucleotide-positive and viral-protein seroconversion was observed in all patients tested and provides evidence of an association between the disease and the presence of this virus. However, there are still many urgent questions that remain to be answered. The association between 2019-nCoV and the disease has not been verified by animal experiments to fulfil the Koch’s postulates to establish a causative relationship between a microorganism and a disease. We do not yet know the transmission routine of this virus among hosts. It appears that the virus is becoming more transmissible between humans. We should closely monitor whether the virus continues to evolve to become more virulent. Owing to a shortage of specific treatments and considering the relatedness of 2019-nCoV to SARS-CoV, some drugs and pre-clinical vaccines against SARS-CoV could probably be used to treat this virus. Finally, considering the wide spread of SARSr-CoV in their natural reservoirs, future research should be focused on active surveillance of these viruses for broader geographical regions. In the long term, broad-spectrum antiviral drugs and vaccines should be prepared for emerging infectious diseases that are caused by this cluster of viruses in the future. Most importantly, strict regulations against the domestication and consumption of wildlife should be implemented.

*Note added in proof:* Since this paper was accepted, the ICTV has designated the virus as SARS-CoV-2^15^; in addition, the WHO has released the official name of the disease caused by this virus, which is COVID-19^16^.

## Methods

### Data reporting

No statistical methods were used to predetermine sample size. The experiments were not randomized and the investigators were not blinded to allocation during experiments and outcome assessment.

### Sample collection

Human samples, including oral swabs, anal swabs, blood and BALF samples were collected by Jinyintan hospital (Wuhan, China) with the consent of all patients and approved by the ethics committee of the designated hospital for emerging infectious diseases. Patients were sampled without gender or age preference unless indicated. For swabs, 1.5 ml DMEM containing 2% FBS was added to each tube. The supernatant was collected after centrifugation at 2,500 rpm, vortexing for 60 s and a standing period of 15–30 min. The supernatant from swabs or BALF (no pre-treatment) was added to either lysis buffer for RNA extraction or to viral transport medium for isolation of the virus. The viral transport medium was composed of Hank’s balanced salt solution (pH 7.4) containing BSA (1%), amphotericin (15 μg ml^−1^), penicillin G (100 units ml^−1^) and streptomycin (50 μg ml^−1^). Serum was separated by centrifugation at 3,000*g* for 15 min within 24 h of collection, followed by inactivation at 56 °C for 1 h, and was then stored at 4 °C until use.

### Virus isolation, cell infection, electron microscopy and neutralization assay

The following cell lines were used for virus isolation in this study: Vero E6 and Huh7 cells, which were cultured in DMEM containing 10% FBS. All cell lines were tested and free of mycoplasma contamination, submitted for species identification and authenticated by morphological evaluation by microscopy. None of the cell lines was on the list of commonly misidentified cell lines (by ICLAC).

Cultured cell monolayers were maintained in their respective medium. The PCR-positive BALF sample from ICU-06 patient was spun at 8,000*g* for 15 min, filtered and diluted 1:2 with DMEM supplemented with 16 μg ml^−1^ trypsin before it was added to the cells. After incubation at 37 °C for 1 h, the inoculum was removed and replaced with fresh culture medium containing antibiotics (see below) and 16 μg ml^−1^ trypsin. The cells were incubated at 37 °C and observed daily for cytopathogenic effects. The culture supernatant was examined for the presence of virus by qRT–PCR methods developed in this study, and cells were examined by immunofluorescence microscopy using the anti-SARSr-CoV Rp3 N antibody that was generated in-house (1:1,000). Penicillin (100 units ml^−1^) and streptomycin (15 μg ml^−1^) were included in all tissue culture media.

Vero E6 cells were infected with the new virus at a multiplicity of infection (MOI) of 0.5 and collected 48 h after infection. Cells were fixed with 2.5% (w/v) glutaraldehyde and 1% osmium tetroxide, dehydrated through a graded series of ethanol concentrations (from 30 to 100%) and embedded with epoxy resin. Ultrathin sections (80 nm) of embedded cells were prepared, deposited onto Formvar-coated copper grids (200 mesh), stained with uranyl acetate and lead citrate, and analysed using a 200-kV Tecnai G2 electron microscope.

The virus neutralization test was carried out in a 96-well plate. The patient serum samples were heat-inactivated by incubation at 56 °C for 1 h before use. The serum samples were diluted to 1:10, 1:20, 1:40 or 1:80, and then an equal volume of virus stock was added and incubated at 37 °C for 60 min in a 5% CO_2_ incubator. Diluted horse anti-SARS-CoV serum or serum samples from healthy individuals were used as control. After incubation, 100 μl mixtures were inoculated onto a monolayer of Vero E6 cells in a 96-well plate for 1 h. Each serum was assessed in triplicate. After removing the supernatant, the plate was washed twice with DMEM medium. Cells were incubated with DMEM supplemented with 2% FBS for 3 days. Subsequently, the cells were checked for cytopathogenic effects.

### RNA extraction and PCR

Whenever commercial kits were used, the manufacturer’s instructions were followed without modification. RNA was extracted from 200 μl of samples with the High Pure Viral RNA kit (Roche). RNA was eluted in 50 μl of elution buffer and used as the template for RT–PCR.

For qPCR analysis, primers based on the *S* gene of 2019-nCoV were designed: RBD-qF1, 5′-CAATGGTTTAACAGGCACAGG-3′; RBD-qR1, 5′-CTCAAGTGTCTGTGGATCACG-3′. RNA extracted as described above was used for qPCR using the HiScript II One Step qRT–PCR SYBR Green Kit (Vazyme Biotech). Conventional PCRs were also performed using the following primer pairs: ND-CoVs-951F, 5′-TGTKAGRTTYCCTAAYATTAC-3′; ND-CoVs-1805R, 5′-ACATCYTGATANARAACAGC-3′. The 20-μl qPCR reaction mix contained 10 μl 2× One Step SYBR Green mix, 1 μl One Step SYBR Green Enzyme mix, 0.4 μl 50× ROX Reference Dye 1, 0.4 μl of each primer (10 μM) and 2 μl template RNA. Amplification was performed as follows: 50 °C for 3 min, 95 °C for 30 s followed by 40 cycles consisting of 95 °C for 10 s and 60 °C for 30 s, and a default melting curve step in an ABI 7500 Real-time PCR machine.

### Serological test

In-house anti-SARSr-CoV IgG and IgM ELISA kits were developed using SARSr-CoV Rp3 N protein as antigen, which shared more than 90% amino acid identity to all SARSr-CoVs^2^. For IgG analyses, MaxiSorp Nunc-immuno 96-well ELISA plates were coated (100 ng per well) overnight with recombinant N protein. Human sera were used at a dilution of 1:20 for 1 h at 37 °C. An anti-human IgG HRP-conjugated monoclonal antibody (Kyab Biotech) was used at a dilution of 1:40,000. The OD value (450–630 nm) was calculated. For IgM analyses, MaxiSorp Nunc-immuno 96-well ELISA plates were coated (500 ng per well) overnight with anti-human IgM (μ chain). Human sera were used at a 1:100 dilution for 40 min at 37 °C, followed by incubation with an anti-Rp3 N HRP-conjugated antibody (Kyab Biotech) at a dilution of 1:4,000. The OD value (450–630 nm) was calculated.

### Examination of ACE2 receptor for 2019-nCoV infection

HeLa cells transiently expressing ACE2 were prepared using Lipofectamine 3000 (Thermo Fisher Scientific) in a 96-well plate; mock-transfected cells were used as controls. 2019-nCoV grown in Vero E6 cells was used for infection at a MOI of 0.5. APN and DPP4 were analysed in the same way. The inoculum was removed after absorption for 1 h and washed twice with PBS and supplemented with medium. At 24 h after infection, cells were washed with PBS and fixed with 4% formaldehyde in PBS (pH 7.4) for 20 min at room temperature. ACE2 expression was detected using a mouse anti-S tag monoclonal antibody and a FITC-labelled goat anti-mouse IgG H&L (Abcam, ab96879). Viral replication was detected using a rabbit antibody against the Rp3 N protein (generated in-house, 1:1,000) and a Cy3-conjugated goat anti-rabbit IgG (1:200, Abcam, ab6939). Nuclei were stained with DAPI (Beyotime). Staining patterns were examined using confocal microscopy on a FV1200 microscope (Olympus).

### High-throughput sequencing, pathogen screening and genome assembly

Samples from patient BALF or from the supernatant of virus cultures were used for RNA extraction and next-generation sequencing (NGS) using BGI MGISEQ2000 and Illumina MiSeq 3000 sequencers. Metagenomic analysis was carried out mainly based on the bioinformatics platform MGmapper (PE_2.24 and SE_2.24). The raw NGS reads were first processed by Cutadapt (v.1.18) with minimum read length of 30 base pairs. BWA (v.0.7.12-r1039) was used to align reads to a local database with a filter hits parameter of 0.8 FMM ((match + mismatch)/read length ≥ fraction] value and minimum alignment score of 30. Parameters for post-processing of assigned reads were set to a minimum size normalized abundance of 0.01, minimum read count of 20 and were otherwise set to default parameters. A local nucleic acid database for human and mammals was used to filter reads of host genomes before mapping reads to the virus database. The results of the metagenomic analysis were displayed as pie charts using Microsoft Office 2010. NGS reads were assembled into genomes using Geneious (v.11.0.3) and MEGAHIT (v.1.2.9). PCR and Sanger sequencing was performed to fill gaps in the genome. 5′-rapid amplification of cDNA ends (RACE) was performed to determine the 5′-end of the genomes using a SMARTer RACE 5′/3′ kit (Takara). Genomes were annotated using the Clone Manager Professional Suite 8 (Sci-Ed Software).

### Phylogenetic analysis

Routine sequence management and analysis was carried out using DNAStar. The sequence alignment of complete genome sequences was performed using MAFFT (v.7.307) with default parameters. The codon alignments of full-length S and RdRp gene sequences were converted from the corresponding protein alignments by PAL2NAL (v.14); the protein alignments were created by Clustal Omega (v.1.2.4) using default parameters. Maximum likelihood phylogenetic trees were generated using RAxML (v.0.9.0) with GTR+G substitution model and 1,000 bootstrap replicates.

### Reporting summary

Further information on research design is available in the Nature Research Reporting Summary linked to this paper.

## Online content

Any methods, additional references, Nature Research reporting summaries, source data, extended data, supplementary information, acknowledgements, peer review information; details of author contributions and competing interests; and statements of data and code availability are available at 10.1038/s41586-020-2012-7.

### Supplementary information

Reporting Summary

## Figures and Tables

**Extended Data Table 1:**
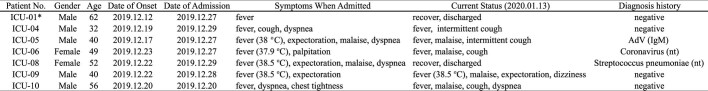
Patient information and diagnosis history

Note, some records are missing. All patients are sellers or deliverymen at the seafood market except ICU-01, whose contact history is unclear. All patients were admitted to intensive care unit (ICU) during the first investigation and were now in stable condition. Blood IgM tests have been performed for the following respiratory pathogens for all patients: *Legionella pneumophilia*, *Mycoplasma pneumoniae*, *Chlamydia pneumoniae*, respiratory syncytial virus, adenovirus, *Rickettsia*, influenza A virus, influenza B virus and parainfluenza virus.

*This patient reported fever on 12 December 2019 and then recovered without medical treatment. He came back to the hospital on 27 December 2019 with a fever. His wife was also ill and admitted to the hospital. Both individuals recovered.

**Extended Data Table 2:**
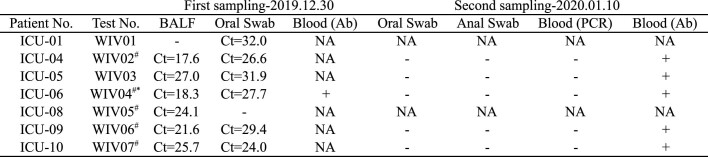
Laboratory results

Samples from two patients (ICU-01 and ICU-08) were not available during the second investigation. They had been discharged from the hospital. We did a serial test for patient ICU-06 on the following dates: 30 December 2019, 31 December 2019, 1 January 2020 and 10 January 2020, corresponding to 7, 8, 9 and 18 days after disease onset (23 December 2019). Molecular and serological (IgM and IgG) virus-detection results for 2019-nCoV are shown. NA, not available.

*Virus isolated.

^#^A full-length genome was obtained.

**Extended Data Table 3:**
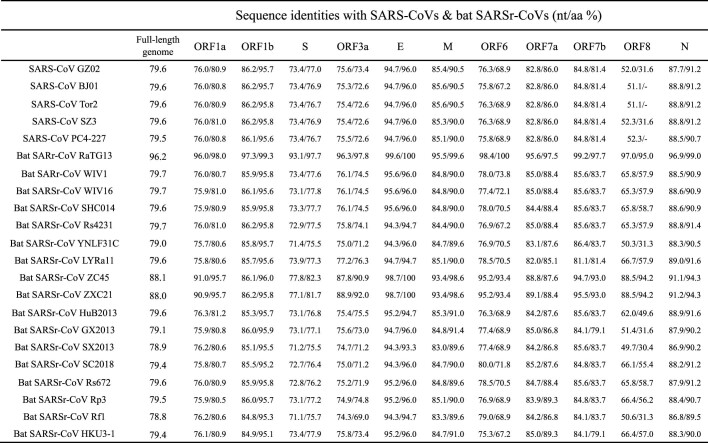
Genomic comparison of 2019-nCoV WIV04 with SARS-CoVs and bat SARSr-CoVs

**Extended Data Table 4:**
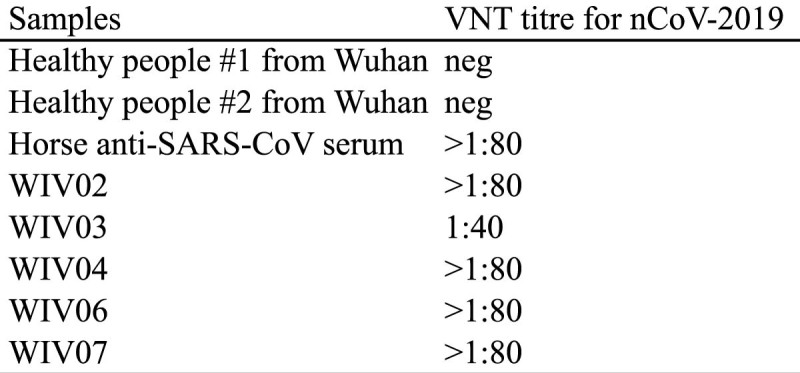
Virus neutralization test of serum samples

Each serum sample was tested in triplicate. Serum samples from two healthy individuals from Wuhan and five patients as well as a horse anti-SARS-CoV anti-serum were used. We used 120 TCID_50_ viruses per well. Serum samples were used at dilutions of 1:10, 1:20, 1:40 and 1:80. neg, negative; VNT, virus neutralization test.

## Data Availability

Sequence data that support the findings of this study have been deposited in GISAID (https://www.gisaid.org/) with accession numbers EPI_ISL_402124, EPI_ISL_402127–EPI_ISL_402130 and EPI_ISL_402131; GenBank with accession numbers MN996527–MN996532; National Genomics Data Center, Beijing Institute of Genomics, Chinese Academy of Sciences (https://bigd.big.ac.cn/databases?lang=en) with accession numbers SAMC133236–SAMC133240 and SAMC133252.

## References

[1] Li W (2005). Bats are natural reservoirs of SARS-like coronaviruses. Science.

[2] Ge X-Y (2013). Isolation and characterization of a bat SARS-like coronavirus that uses the ACE2 receptor. Nature.

[3] Yang L (2013). Novel SARS-like betacoronaviruses in bats, China, 2011. Emerg. Infect. Dis..

[4] Hu B (2017). Discovery of a rich gene pool of bat SARS-related coronaviruses provides new insights into the origin of SARS coronavirus. PLoS Pathog..

[5] Menachery VD (2015). A SARS-like cluster of circulating bat coronaviruses shows potential for human emergence. Nat. Med..

[6] Menachery VD (2016). SARS-like WIV1-CoV poised for human emergence. Proc. Natl Acad. Sci. USA.

[7] Wang N (2018). Serological evidence of bat SARS-related coronavirus infection in humans, China. Virol. Sin..

[8] Drosten C (2003). Identification of a novel coronavirus in patients with severe acute respiratory syndrome. N. Engl. J. Med..

[9] Zaki AM, van Boheemen S, Bestebroer TM, Osterhaus ADME, Fouchier RAM (2012). Isolation of a novel coronavirus from a man with pneumonia in Saudi Arabia. N. Engl. J. Med..

[10] Cui J, Li F, Shi ZL (2019). Origin and evolution of pathogenic coronaviruses. Nat. Rev. Microbiol..

[11] Fan Y, Zhao K, Shi Z-L, Zhou P (2019). Bat coronaviruses in China. Viruses.

[12] Wuhan Municipal Health Commission. Press statement related to novel coronavirus infection (in Chinese) http://wjw.wuhan.gov.cn/front/web/showDetail/2020012709194 (2020).

[13] Poon LL (2005). Identification of a novel coronavirus in bats. J. Virol..

[14] Li W (2003). Angiotensin-converting enzyme 2 is a functional receptor for the SARS coronavirus. Nature.

[15] Gorbalenya, A. E. et al. Severe acute respiratory syndrome-related coronavirus — the species and its viruses, a statement of the Coronavirus Study Group. Preprint at https://www.biorxiv.org/content/10.1101/2020.02.07.937862v1 (2020).

[16] WHO. WHO Director-General’s remarks at the media briefing on 2019-nCoV on 11 February 2020. https://www.who.int/dg/speeches/detail/who-director-general-s-remarks-at-the-media-briefing-on-2019-ncov-on-11-february-2020 (WHO, 11 February 2020).

