# Supplementary material for: A pneumonia outbreak associated with a new coronavirus of probable bat origin
Source: Nature. 2020 Feb 3;579(7798):270–3. doi: 10.1038/s41586-020-2012-7 (PMC7095418; doi:10.1038/s41586-020-2012-7)
Supplement: Supplementary file 1 — Reporting Summary [file 41586_2020_2012_MOESM1_ESM.pdf]

nature research

Corresponding author(s): Zheng-Li Shi

Last updated by author(s): Feb 21, 2020

Reporting Summary

Nature Research wishes to improve the reproducibility of the work that we publish. This form provides structure for consistency and transparency in reporting. For further information on Nature Research policies, see Authors & Referees and the Editorial Policy Checklist.

Statistics

For all statistical analyses, confirm that the following items are present in the figure legend, table legend, main text, or Methods section.

n/a	Confirmed	
☐	☒	The exact sample size (*n*) for each experimental group/condition, given as a discrete number and unit of measurement
☐	☒	A statement on whether measurements were taken from distinct samples or whether the same sample was measured repeatedly
☒	☐	The statistical test(s) used AND whether they are one- or two-sided<br>*Only common tests should be described solely by name; describe more complex techniques in the Methods section.*
☒	☐	A description of all covariates tested
☒	☐	A description of any assumptions or corrections, such as tests of normality and adjustment for multiple comparisons
☒	☐	A full description of the statistical parameters including central tendency (e.g. means) or other basic estimates (e.g. regression coefficient) AND variation (e.g. standard deviation) or associated estimates of uncertainty (e.g. confidence intervals)
☒	☐	For null hypothesis testing, the test statistic (e.g. *F*, *t*, *r*) with confidence intervals, effect sizes, degrees of freedom and *P* value noted<br>*Give P values as exact values whenever suitable.*
☒	☐	For Bayesian analysis, information on the choice of priors and Markov chain Monte Carlo settings
☒	☐	For hierarchical and complex designs, identification of the appropriate level for tests and full reporting of outcomes
☒	☐	Estimates of effect sizes (e.g. Cohen's *d*, Pearson's *r*), indicating how they were calculated

Our web collection on statistics for biologists contains articles on many of the points above.

Software and code

Policy information about availability of computer code

Data collection	No software was used.
Data analysis	BWA (v0.712-r1039), Cutadapt (v1.18), Geneious (v11.0.3), MEGAHIT (v1.2.9), Clone Manager Professional Suite 8, MAFFT (v7.307), MGmapper (PE2.24 and SE2.24), PAL2NAL (version 14), Clustal Omega (version 1.2.4), RAxML (version 0.9.0)

For manuscripts utilizing custom algorithms or software that are central to the research but not yet described in published literature, software must be made available to editors/reviewers. We strongly encourage code deposition in a community repository (e.g. GitHub). See the Nature Research guidelines for submitting code & software for further information.

Data

Policy information about availability of data

All manuscripts must include a data availability statement. This statement should provide the following information, where applicable:

- Accession codes, unique identifiers, or web links for publicly available datasets
- A list of figures that have associated raw data
- A description of any restrictions on data availability

Sequence data that support the findings of this study have been deposited in GISAID with the accession no. EPI_ISL_402124 and EPI_ISL_402127-402131.

Field-specific reporting

Please select the one below that is the best fit for your research. If you are not sure, read the appropriate sections before making your selection.

☒ Life sciences ☐ Behavioural & social sciences ☐ Ecological, evolutionary & environmental sciences

For a reference copy of the document with all sections, see nature.com/documents/nr-reporting-summary-flat.pdf

Life sciences study design

All studies must disclose on these points even when the disclosure is negative.

Sample size	Samples of seven pneumonia patients are available from the clinical hospital to be sent to Wuhan Institute of Virology for pathogen identification. The coronavirus genome sequences were obtained from 5 different patients and shared >99.9% identity, suggesting they were infected by the same virus. Therefore, the sample size is sufficient for conducting the following study which aims to identify and characterize the causative agent of this pneumonia outbreak.
Data exclusions	No data excluded
Replication	The authors guarantee the findings are reliably reproducible. At least three independent experiments were performed, which was stated in the text.
Randomization	Samples were chosen randomly.
Blinding	We were blinded when choosing samples.

Reporting for specific materials, systems and methods

We require information from authors about some types of materials, experimental systems and methods used in many studies. Here, indicate whether each material, system or method listed is relevant to your study. If you are not sure if a list item applies to your research, read the appropriate section before selecting a response.

Materials & experimental systems

n/a	Involved in the study	
☐	☒	Antibodies
☐	☒	Eukaryotic cell lines
☒	☐	Palaeontology
☒	☐	Animals and other organisms
☐	☒	Human research participants
☒	☐	Clinical data

Methods

n/a	Involved in the study	
☒	☐	ChIP-seq
☒	☐	Flow cytometry
☒	☐	MRI-based neuroimaging

Antibodies

Antibodies used	1. SARSr-CoV Rp3 NP antibody made in house; 2. Anti-Human IgG-HRP conjugated monoclonal antibody (Kyab Biotech Co., Ltd,Wuhan, China, dilution: 1:40000); 3. Anti-Rp3 NP-HRP conjugated (Kyab Biotech Co.,Ltd, Wuhan, China, dilution: 1:4000); 4.FITC-labelled goat anti-mouse IgG H&L (Abcam, ab96879, dlilution 1:100); 5. cyanin 3-conjugated goat anti-rabbit IgG (Abcam,ab6939, dilution: 1:200); 6. mouse anti-S tag monoclonal antibody made in house
Validation	The house-made SARSr-CoV Rp3 NP antibodies and anti-S tag monoclonal antibody were validated in a WB. The cy3-conjugated anti-rabbit IgGs were validated in IFA. The FITC-labelled goat anti-mouse IgG H&L was validated in IHC.

Eukaryotic cell lines

Policy information about cell lines

Cell line source(s)	1. African green monkey origin, Vero E6 cell; 2. Human lung cell Huh7 ; 3. Human HeLa cells. All cell lines werefrom ATCC.
Authentication	All monkey and human cells were from ATCC with authentication. The authentication was performed by microscope morphology check, growth curve analysis or identity verification with STR analysis (for human cell lines).
Mycoplasma contamination	We confirm that all cells were tested as mycoplasma negative.
Commonly misidentified lines (See ICLAC register)	No commonly misidentified cell lines were used.

Human research participants

Policy information about studies involving human research participants

Population characteristics	Participants were all 2019-nCoV infected patients.
Recruitment	Samples were sent to Wuhan Institute of Virology by hospital for pathogen identification.
Ethics oversight	Wuhan Jinyintan Hospital (the co-authored institution)

Note that full information on the approval of the study protocol must also be provided in the manuscript.
